# Combined Aerobic and Resistance Exercise Program in Adults: Endocrine and Metabolic Adaptations

**DOI:** 10.3390/life16081365

**Published:** 2026-08-19

**Authors:** Leopoldo Ferrante, Francesca Latino, Giulia Amato, Maria Giovanna Tafuri

**Affiliations:** 1 Department of Economics, Law, Cybersecurity and Sports Sciences, University of Naples Parthenope, 80035 Nola, Italy; lferrante@uniparthenope.it; 2 Department of Biological and Environmental Sciences and Technologies, University of Salento, 73100 Lecce, Italy; 3 Department of Medical, Human Movement and Well-Being Sciences, University of Naples Parthenope, Via Medina 40, 80133 Napoli, Italy; giulia.amato@uniparthenope.it; 4 Department of Literary, Linguistic and Philosophical Studies, Pegaso University, 80143 Naples, Italy; mariagiovanna.tafuri@unipegaso.it

**Keywords:** combined exercise, endocrine system, insulin sensitivity, cortisol, growth hormone, lipid metabolism

## Abstract

Background: Physical exercise induces coordinated endocrine and metabolic adaptations that contribute to the regulation of glucose homeostasis, lipid metabolism, stress responses, and anabolic processes. However, the integrated effects of combined aerobic and resistance exercise on multiple endocrine and metabolic outcomes require further investigation. This study aimed to evaluate the effects of a 12-week combined aerobic and resistance exercise program on endocrine and metabolic parameters in normal-weight and mildly overweight adults. Methods: A total of 60 adults were randomly allocated to an exercise group (EX, *n* = 30) or a control group (CON, *n* = 30). The EX-group completed a supervised 12-week combined exercise program consisting of three 60 min sessions per week, including aerobic exercise and multi-joint resistance training, whereas the CON-group maintained their habitual physical activity levels. Endocrine parameters, including insulin, glucagon, cortisol, growth hormone (GH), thyroid hormones, and sex-specific hormones, as well as fasting glucose, lipid profile, and homeostasis model assessment of insulin resistance (HOMA-IR), were assessed at baseline and after 12 weeks. Results: Compared with the control condition, the exercise program was associated with favorable changes in endocrine and metabolic profiles. The EX-group showed reductions in fasting insulin, cortisol, fasting glucose, and HOMA-IR, together with increased GH levels. Favorable changes were also observed in the lipid profile, including reductions in total cholesterol, low-density lipoprotein cholesterol (LDL), and triglycerides and an increase in high-density lipoproteins cholesterol (HDL). In the male subgroup, total testosterone increased following the intervention, whereas estradiol changes in the female subgroup were more limited. Changes in insulin were associated with changes in glucose regulation and lipid parameters, while GH and cortisol changes showed additional associations with metabolic outcomes. Conclusions: These findings support the role of structured combined exercise as a non-pharmacological strategy for promoting endocrine and metabolic health and preventing metabolic dysfunction.

## 1. Introduction

Physical exercise is a complex biological stimulus capable of producing broad benefits across multiple dimensions of human health and functioning, ranging from physiological well-being to cognitive and psychological outcomes [1,2,3]. Among its systemic effects, exercise can modulate neuroendocrine and metabolic processes in a coordinated manner, thereby contributing to the regulation of energy homeostasis and physiological adaptation [3,4,5]. Muscle contractile activity induces acute endocrine responses and chronic adaptations involving the hypothalamic–pituitary–adrenal (HPA) axis, the somatotropic axis, the gonadal axis, thyroid function and insulin–glucagon regulation, with relevant implications for metabolic health [6].

During acute exercise, the activation of the HPA axis leads to an increase in cortisol secretion proportional to the intensity and duration of the effort, promoting hepatic gluconeogenesis, lipolysis and amino acid mobilization. At the same time, the increase in plasma catecholamines (adrenaline and noradrenaline) stimulates glycogenolysis and lipolysis, optimizing the availability of energy substrates. Growth hormone (GH) shows a marked pulsatile increase during high-intensity exercise, with lipolytic and anabolic effects mediated in part by insulin-like growth factor 1 (IGF-1). These changes represent physiological adaptations aimed at maintaining glycemic balance and muscle performance [7].

A key element that has emerged in recent years is the recognition of skeletal muscle as an endocrine organ. Muscle contraction induces the release of myokines, including interleukin-6 (IL-6), irisin, myostatin, and brain-derived neurotrophic factor (BDNF), which exert autocrine, paracrine, and endocrine effects on the liver, adipose tissue, pancreas, and central nervous system. In particular, muscle-derived IL-6 plays a different role than pro-inflammatory IL-6, contributing to the regulation of lipid metabolism and the improvement of insulin sensitivity [8].

Chronic exercise results in structural endocrine adaptations characterized by improved insulin sensitivity through insulin-dependent and insulin-independent mechanisms (AMPK and CaMK-mediated GLUT4 translocation), reduction in compensatory hyperinsulinemia, and modulation of the balance between leptin and adiponectin [9,10]. These effects are particularly relevant in the prevention and treatment of metabolic syndrome and type 2 diabetes mellitus.

Additional evidence suggests that regular training modulates basal secretion and HPA axis responsiveness, contributing to improved stress resilience and a reduction in chronic low-grade inflammatory status [11]. Similarly, resistance and high-intensity exercise differentially affect testosterone and GH secretion, with implications for body composition and protein turnover. Endocrine responses are also modulated by variables such as age, biological sex, nutritional status, nutrient timing and training status [12].

In the context of clinical and sports nutrition, the interaction between physical exercise and endocrine function plays a central role. Protein intake, carbohydrate availability, and energy balance can influence insulin, GH, and cortisol response, helping to modulate exercise-induced metabolic and mitochondrial adaptations [13,14]. In this context, it is particularly relevant to understand the extent to which a structured exercise program, even in the absence of a specific nutritional intervention, can promote coordinated adaptations at the endocrine and metabolic level [15]. The rationale for combining aerobic and resistance exercise lies in the complementary physiological adaptations elicited by these two exercise modalities. Aerobic exercise primarily promotes cardiorespiratory and oxidative adaptations and contributes to the regulation of glucose and lipid metabolism, whereas resistance exercise provides a distinct stimulus for skeletal muscle adaptation, with potential effects on glucose disposal and endocrine regulation. Their combination may therefore provide a broader physiological stimulus capable of concurrently targeting multiple pathways involved in endocrine and metabolic homeostasis [16,17,18,19].

In light of these evidences, the present study aims to evaluate the effects of a combined 12-week aerobic and resistance exercise program on the endocrine and metabolic structure of normal weight or slightly overweight adults. In particular, the study intends to examine the changes in the main indicators of endocrine regulation, including insulin, glucagon, cortisol, growth hormone, sex hormones and thyroid hormones, as well as the changes in metabolic parameters related to glucose homeostasis, insulin sensitivity and lipid profile.

## 2. Materials and Methods

### 2.1. Study Design

The study was designed as a randomized, controlled, parallel-group, longitudinal experimental trial lasting 12 weeks, aimed at evaluating the impact of a combined aerobic and resistance exercise program on endocrine profile and metabolic parameters in normal weight or mildly overweight adults.

Participants were randomly assigned in a 1:1 ratio to either the intervention group (Exercise Group, EX) or the control group (Control Group, CON) using a computer-generated random allocation sequence. The sequence was generated by a researcher who was not involved in participant recruitment and remained inaccessible to the investigators responsible for enrolment until the time of group assignment, thereby ensuring allocation concealment. Assessments were made at baseline (T0) and at the end of the intervention (T1, 12 weeks).

The study design was developed in accordance with CONSORT recommendations for randomized trials and in line with ethical guidelines for human clinical research. The study was conducted in accordance with the Declaration of Helsinki. All participants provided written informed consent prior to enrollment. The protocol has been approved by the competent Ethics Committee.

### 2.2. Participants

Participants were recruited on a voluntary basis and underwent a preliminary eligibility assessment based on predefined inclusion and exclusion criteria. Adults aged 18 to 65 years with a body mass index (BMI) between 18.5 and 30 kg/m^2^, sedentary or moderately active, without a documented diagnosis of endocrine disorders, including diabetes mellitus, hypothyroidism or Cushing’s syndrome, and suitable for moderate-vigorous intensity exercise following a preliminary clinical evaluation were considered eligible.

Exclusion criteria included pregnancy or breastfeeding, taking medications potentially capable of affecting endocrine function, including systemic corticosteroids, hormone replacement therapy or antiandrogens, as well as the presence of uncontrolled cardiovascular disease or any other clinical condition incompatible with participation in the exercise program.

The sample size was estimated as a priori by power analysis [20] for a repeated-measures design with two groups (EX vs. CON) and two evaluation times (T0 vs. T1), taking as the primary endpoint the change in the HOMA-IR index. In the absence of specific preliminary data for the study population, the estimate was based on an expected moderate-amplitude effect for time-× group interaction (Cohen’s f = 0.25). Assuming a significance level α = 0.05, a statistical power (1 − β) = 0.80, a correlation between repeated measures equal to r = 0.50 and a non-sphericity correction parameter ε = 1, the analysis, conducted by G*Power, version 3.1.9.7 (Universität Düsseldorf, Germany), indicated a minimum total number of 34 participants, corresponding to 17 subjects per group. Considering an expected drop-out rate of 15–20%, the minimum recruitment target was increased to 40–42 participants.

**Figure 1:**
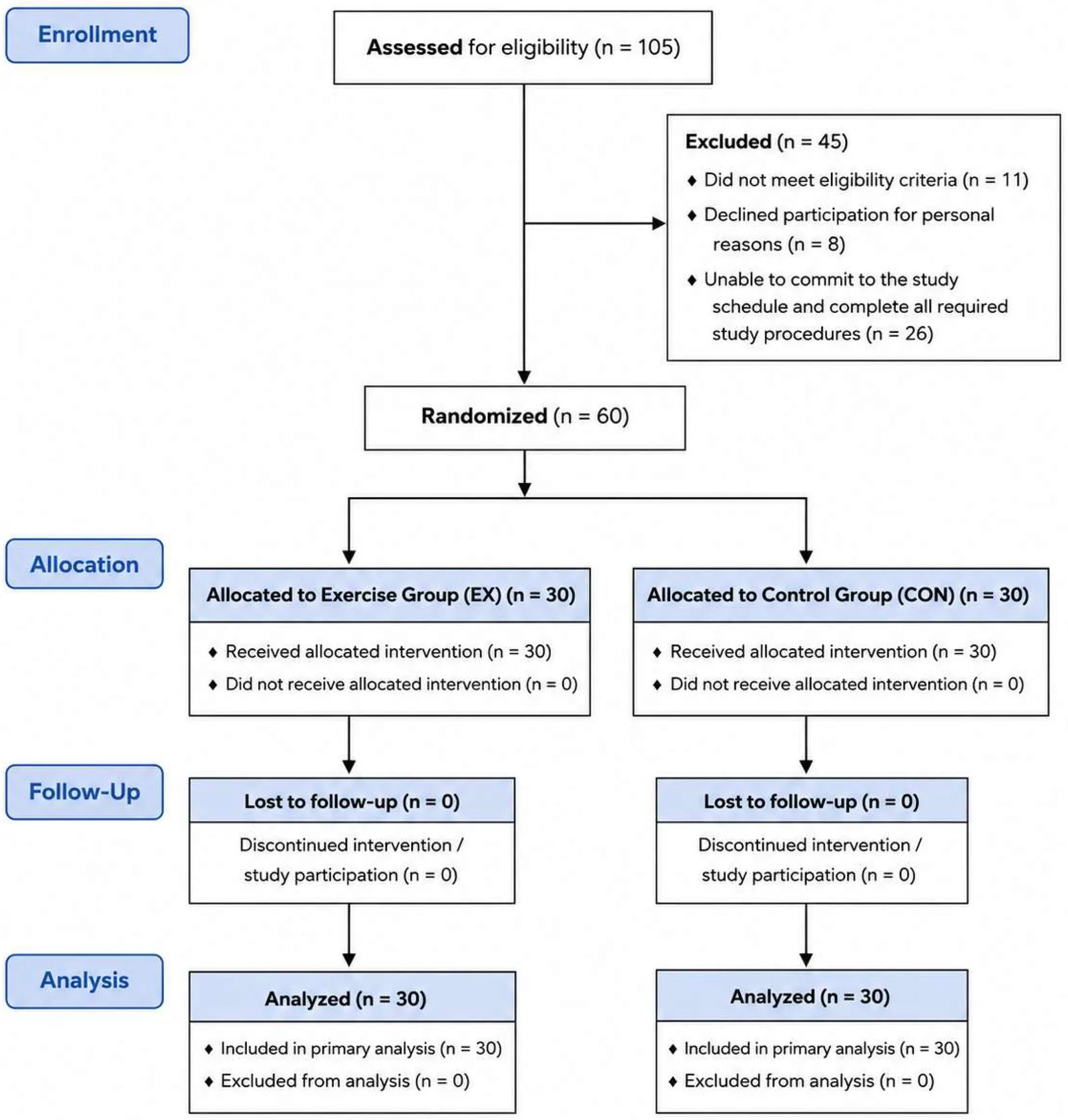
Flow diagram of participant recruitment, allocation, follow-up, and analysis.

A total of 105 individuals were assessed for eligibility. Of these, 45 were excluded before randomization: 11 did not meet the eligibility criteria, 8 declined participation for unspecified personal reasons, and 26 were unable to commit to the study schedule and complete all required study procedures. The remaining 60 participants were included in the study, a number higher than the minimum threshold estimated by the power analysis, and were subsequently randomly assigned in a 1:1 ratio to either the exercise group (EX, *n* = 30) or the control group (CON, *n* = 30). Participant flow through recruitment, allocation, follow-up, and analysis is presented in Figure 1.

### 2.3. Procedures

After recruitment, participants underwent a preliminary assessment to verify compliance with the eligibility criteria and clinical suitability for participation in the study. Eligible subjects received detailed information about the research purposes and procedures and, after providing written informed consent, underwent baseline assessments (T0).

Assessment procedures were conducted in a standardized manner at baseline (T0) and at the end of 12 weeks (T1). On both occasions, the endocrine and metabolic assessments required by the protocol were carried out. Blood samples were taken in the morning, between 07:30 and 09:00, after an overnight fast of at least 10 to 12 h, in order to limit the variability associated with circadian rhythms and recent nutrient intake. Participants were also instructed to follow a standardized dietary intake on the day preceding each blood collection and to avoid strenuous physical exercise for 48 h before sampling. For female participants, menstrual cycle phase was documented at baseline, and post-intervention blood sampling was scheduled during the corresponding phase of the menstrual cycle for each individual participant. This procedure was adopted to minimize intra-individual variability in hormone concentrations related to menstrual cycle fluctuations.

After baseline assessments, participants were randomly assigned, with a 1:1 allocation ratio, to either the exercise group (EX) or the control group (CON). During the next 12 weeks, the EX-group participated in a supervised program of combined aerobic and resistance exercise, carried out three times a week, while the CON-group maintained their usual level of physical activity and were asked not to undertake new structured exercise programs during the study period. Participants in both groups were instructed to maintain their habitual dietary patterns throughout the 12-week study period and to avoid intentional changes in their usual dietary intake.

At the end of the 12 weeks, participants underwent post-intervention assessments (T1), repeating the same measurement procedures carried out at baseline and under the same standardized conditions. This procedure made it possible to evaluate the changes in endocrine and metabolic parameters over time and to compare their evolution between the exercise group and the control group.

### 2.4. Exercise Intervention

The training protocol was structured according to the principles of the American College of Sports Medicine, involving a combination of aerobic and resistance exercise.

The EX-group had three supervised weekly sessions, each lasting 60 min, for 12 consecutive weeks. Each session included:

- 30 min of aerobic activity (brisk walking, jogging or cycling) at 60–80% of your maximum heart rate (HRmax), calculated according to the standard formula (220 − age);
- 30 min of multi-joint resistance exercises (3 sets of 8–12 repetitions per major muscle group, intensity equal to 60–70% of estimated 1-RM). Training progression was implemented gradually throughout the 12-week intervention. For the aerobic component, participants initially exercised at an intensity close to 60% of HRmax, with intensity progressively increased toward 70–80% of HRmax according to individual tolerance and ability to complete the prescribed exercise duration. For the resistance component, the initial load corresponded to approximately 60–70% of estimated 1-RM. Loads were progressively increased when participants were able to correctly complete three sets of 12 repetitions with appropriate exercise technique, thereby maintaining the prescribed repetition range as training adaptations occurred. All exercise sessions were supervised, and attendance was recorded at each session. Adherence was calculated as the percentage of completed sessions relative to the 36 sessions prescribed over the 12-week intervention.

The CON-group maintained their usual level of physical activity, with an indication not to undertake new structured programs during the study period.

### 2.5. Measures

#### 2.5.1. Blood Sampling

Blood samples were taken at baseline and after 12 weeks, in the morning (07:30–09:00), after an overnight fast of at least 10–12 h, to minimize circadian variability of hormones.

#### 2.5.2. Hormonal Analysis

Endocrine parameters analyzed included:

- Insulin and glucagon, determined by enzyme immunoassay (ELISA) (Mercodia AB, Uppsala, Sweden).
- Serum cortisol, measured by chemiluminescent immunoassay (DiaSorin S.p.A., Saluggia, Italy).
- GH, by immunoradiometric method (Beckman Coulter, Brea, CA, USA).
- Total testosterone or estradiol (depending on biological sex), via validated immunoassay (Roche Diagnostics, Mannheim, Germany).
- Thyroid hormones, including total triiodothyronine (T3) and total thyroxine (T4), were determined by chemiluminescent immunoassay (CLIA) (Roche Diagnostics, Mannheim, Germany). Total rather than free thyroid hormone concentrations were selected to provide an overall assessment of circulating thyroid hormone levels within the broader endocrine profile examined in this study, which was designed to characterize exercise-related endocrine adaptations rather than to provide a detailed clinical assessment of thyroid function.

#### 2.5.3. Metabolic Parameters

Fasting plasma glucose, complete lipid profile (total cholesterol, HDL, LDL, triglycerides) and HOMA-IR index were evaluated, calculated according to the formula:

HOMA-IR = [Insulin (μU/mL) × Glucose (mg/dL)]/405.

#### 2.5.4. Physical Activity Questionnaire-Short Form (IPAQ-SF)

Habitual physical activity was assessed using the International Physical Activity Questionnaire-Short Form (IPAQ-SF), which records the frequency and duration of walking and moderate- and vigorous-intensity physical activity during the previous seven days. Physical activity was quantified according to the IPAQ scoring protocol and expressed as MET-hours/week. The same questionnaire was administered at T1 to verify maintenance of habitual physical activity levels in the CON group during the intervention period.

### 2.6. Statistical Analysis

Statistical analysis was conducted using IBM SPSS Statistics, version 30.0 (IBM Corp., Armonk, NY, USA). The normality of the distribution of continuous variables was verified by the Shapiro–Wilk test and the inspection of the related descriptive parameters. Continuous data were expressed as mean ± standard deviation, while categorical variables were reported as absolute frequencies and percentages.

Differences between the EX- group and the CON-group in continuous variables at baseline were assessed by independent sample t-tests. In the CON group, habitual physical activity levels measured by the IPAQ-SF at T0 and T1 were compared using a paired-samples t-test to verify whether physical activity remained stable during the 12-week study period. The effects of the intervention on endocrine and metabolic parameters were analyzed by repeated measures ANOVA 2 × 2, considering time (T0 vs. T1) as an intra-subject factor and group (EX vs. CON) as an inter-subject factor. The main effects of time and group were examined, as well as the Time × Group interaction, considering the effect of greatest interest to assess the differences in the variation in outcomes between the two groups during the intervention.

For significant effects, post hoc analyses were conducted to examine changes between T0 and T1 within each group and differences between groups at individual assessment time points, with Bonferroni adjustment applied to account for multiple comparisons. Effect magnitude was expressed as partial eta squared (ηp^2^), with values of approximately 0.01, 0.06, and 0.14 interpreted as small, moderate, and large effects, respectively. In addition, 95% confidence intervals (95% CIs) were calculated for the principal effect estimates to provide information on the precision of the observed effects.

In addition, exploratory sex-based analyses were conducted for fasting glucose, lipid outcomes, and HOMA-IR by including biological sex as a between-subject factor in the repeated-measures model and examining the Time × Group × Sex interaction. Given the sample size within the sex-specific subgroups, these analyses were considered exploratory.

The associations between changes in hormonal parameters and those in metabolic parameters were analyzed by Pearson correlation coefficients. For each variable, individual changes were calculated as the difference between the post-intervention value and the baseline value (Δ = T1 − T0). All statistical tests were conducted at two tails, and the level of statistical significance was set at *p* < 0.05. All randomized participants were analyzed according to their originally assigned groups, consistent with the intention-to-treat principle. As all 60 randomized participants completed both T0 and T1 assessments and complete outcome data were available, no imputation of missing data was required.

## 3. Results

### 3.1. Demographic and Baseline Characteristics

A total of 60 participants were randomized, with 30 allocated to the exercise group (EX) and 30 to the control group (CON). All randomized participants completed the 12-week study and both T0 and T1 assessments; therefore, no participants withdrew or were lost to follow-up after randomization. All 60 randomized participants were included in the final analysis. The demographic and baseline characteristics of the participants are presented in Table 1. The two groups showed similar baseline characteristics in terms of age, body mass index (BMI), and habitual physical activity level. The sex distribution was also comparable between groups, with 15 men and 15 women in the EX-group and 14 men and 16 women in the CON-group. Adherence to the exercise intervention was high, with participants in the EX-group completing 96% of the 36 prescribed exercise sessions. No adverse events related to the exercise intervention were reported during the study period. In the CON group, habitual physical activity remained stable over the 12-week study period, with no significant difference between T0 (8.3 ± 2.1 MET-hours/week) and T1 (8.4 ± 2.2 MET-hours/week; t = 0.31, *p* = 0.759).

### 3.2. Changes in Endocrine Parameters

Endocrine parameters were assessed at baseline (T0) and after the 12-week intervention period (T1). The repeated-measures analyses revealed significant Time × Group interactions for fasting insulin, T3, cortisol, and GH, indicating different patterns of change between the EX and CON groups over the intervention period. The corresponding results are presented in Table 2.

A significant Time × Group interaction was observed for fasting insulin (F = 14.82, *p* < 0.001, ηp^2^ = 0.204). Insulin concentrations decreased from 12.5 ± 4.2 μU/mL at T0 to 8.9 ± 3.5 μU/mL at T1 in the EX-group, whereas only a minimal change was observed in the CON group. Post hoc analyses confirmed a significant reduction from T0 to T1 in the EX-group (*p* < 0.001), while no significant change was observed in the CON group (*p* = 0.631). At T1, fasting insulin concentrations were significantly lower in the EX-group than in the CON-group (*p* = 0.002).

A significant Time × Group interaction was also observed for cortisol (F = 5.89, *p* = 0.018, ηp^2^ = 0.092). Cortisol concentrations decreased from 16.2 ± 4.3 μg/dL to 14.5 ± 3.9 μg/dL in the EX-group, while remaining substantially stable in the CON-group. Post hoc analyses showed a significant reduction from T0 to T1 in the EX-group (*p* = 0.011), whereas no significant within-group change was observed in CON (*p* = 0.842).

GH concentrations showed a significant Time × Group interaction (F = 18.64, *p* < 0.001, ηp^2^ = 0.243). In the EX-group, GH increased from 1.5 ± 0.4 ng/mL at baseline to 2.3 ± 0.5 ng/mL after the intervention, while concentrations remained unchanged in the CON group. Post hoc analyses confirmed a significant increase from T0 to T1 in EX (*p* < 0.001), whereas no significant change was observed in CON (*p* = 0.918).

Regarding thyroid hormones, a significant Time × Group interaction was observed for total T3 (F = 4.76, *p* = 0.033, ηp^2^ = 0.076). Total T3 concentrations increased from 1.1 ± 0.2 ng/mL to 1.2 ± 0.2 ng/mL in the EX-group, whereas they remained stable in the CON group. In contrast, no significant Time × Group interactions were observed for glucagon (F = 0.41, *p* = 0.525, ηp^2^ = 0.007) or total T4 (F = 1.12, *p* = 0.294, ηp^2^ = 0.019).

Sex hormones were analyzed separately according to biological sex. Total testosterone was assessed in male participants, whereas estradiol was assessed in female participants. In men, a significant Time × Group interaction was observed for total testosterone (F = 4.21, *p* = 0.050, ηp^2^ = 0.135). Testosterone concentrations increased from 450 ± 120 ng/dL to 520 ± 130 ng/dL in the EX-group, with a significant within-group increase (*p* = 0.041), whereas values remained substantially stable in the CON group (*p* = 0.768). In women, estradiol concentrations increased modestly from 60.5 ± 15.3 pg/mL to 65.2 ± 15.8 pg/mL in the EX-group, while remaining stable in the CON group. However, the Time × Group interaction was not statistically significant (F = 1.36, *p* = 0.253, ηp^2^ = 0.046), and the within-group change in EX did not reach statistical significance (*p* = 0.118).

### 3.3. Changes in Metabolic Parameters

Metabolic parameters were assessed at baseline and after 12 weeks to examine changes in glucose homeostasis, insulin sensitivity, and lipid profile (Table 3). Significant Time × Group interactions were observed for fasting glucose, HOMA-IR, total cholesterol, HDL cholesterol, LDL cholesterol, and triglycerides, indicating a more favorable pattern of metabolic change in the EX-group compared with the CON group.

Fasting glucose showed a significant Time × Group interaction (F = 10.96, *p* = 0.002, ηp^2^ = 0.159). In the EX-group, fasting glucose decreased from 98.2 ± 10.1 mg/dL at T0 to 90.5 ± 9.3 mg/dL at T1, whereas values remained substantially unchanged in the CON group. Post hoc analyses confirmed a significant T0–T1 reduction in EX (*p* = 0.001), while no significant change was observed in CON (*p* = 0.692).

A similar pattern was observed for insulin resistance. HOMA-IR showed a significant Time × Group interaction (F = 9.68, *p* = 0.003, ηp^2^ = 0.143). HOMA-IR decreased from 3.1 ± 1.2 to 2.5 ± 1.0 in the EX-group, whereas only a minimal change was observed in the CON group. Post hoc analyses confirmed a significant improvement in the EX-group (*p* = 0.002), while no significant within-group change occurred in CON (*p* = 0.584).

Significant changes were also observed in the lipid profile. Total cholesterol showed a significant Time × Group interaction (F = 6.84, *p* = 0.011, ηp^2^ = 0.105), decreasing from 200.5 ± 25.3 mg/dL to 185.2 ± 22.8 mg/dL in the EX-group. Post hoc analysis confirmed a significant reduction in EX (*p* = 0.009), whereas no significant change was observed in CON (*p* = 0.711).

HDL cholesterol showed a significant Time × Group interaction (F = 5.21, *p* = 0.026, ηp^2^ = 0.082). HDL concentrations increased from 48.5 ± 10.2 mg/dL to 52.1 ± 10.5 mg/dL in the EX-group, with a significant T0–T1 change (*p* = 0.021), whereas values remained stable in CON (*p* = 0.817).

A significant Time × Group interaction was also observed for LDL cholesterol (F = 5.77, *p* = 0.020, ηp^2^ = 0.090). LDL concentrations decreased from 120.3 ± 20.4 mg/dL to 110.2 ± 18.7 mg/dL in the EX-group. Post hoc analysis showed a significant reduction in EX (*p* = 0.016), while no significant change was observed in CON (*p* = 0.764).

Finally, triglycerides showed a significant Time × Group interaction (F = 7.92, *p* = 0.007, ηp^2^ = 0.120). In the EX-group, triglyceride concentrations decreased from 150.2 ± 35.6 mg/dL at T0 to 130.5 ± 30.2 mg/dL at T1. The reduction was significant in EX (*p* = 0.006), whereas no significant change was observed in the CON group (*p* = 0.836).

Overall, these findings indicate that the 12-week combined exercise intervention was associated with favorable changes in glucose regulation, insulin sensitivity, and lipid metabolism.

### 3.4. Exploratory Sex-Based Analysis

Exploratory sex-based analyses were performed to assess whether intervention-related changes in glucose and lipid outcomes differed between men and women. No significant Time × Group × Sex interactions were observed for fasting glucose (F = 0.84, *p* = 0.364, ηp^2^ = 0.014), total cholesterol (F = 0.57, *p* = 0.454, ηp^2^ = 0.010), HDL cholesterol (F = 1.12, *p* = 0.295, ηp^2^ = 0.019), LDL cholesterol (F = 0.73, *p* = 0.397, ηp^2^ = 0.013), triglycerides (F = 1.36, *p* = 0.248, ηp^2^ = 0.023), or HOMA-IR (F = 0.91, *p* = 0.344, ηp^2^ = 0.016). These findings provided no evidence of sex-dependent differences in intervention-related changes in the metabolic outcomes examined.

### 3.5. Correlations Between Changes in Endocrine and Metabolic Parameters

**Figure 2:**
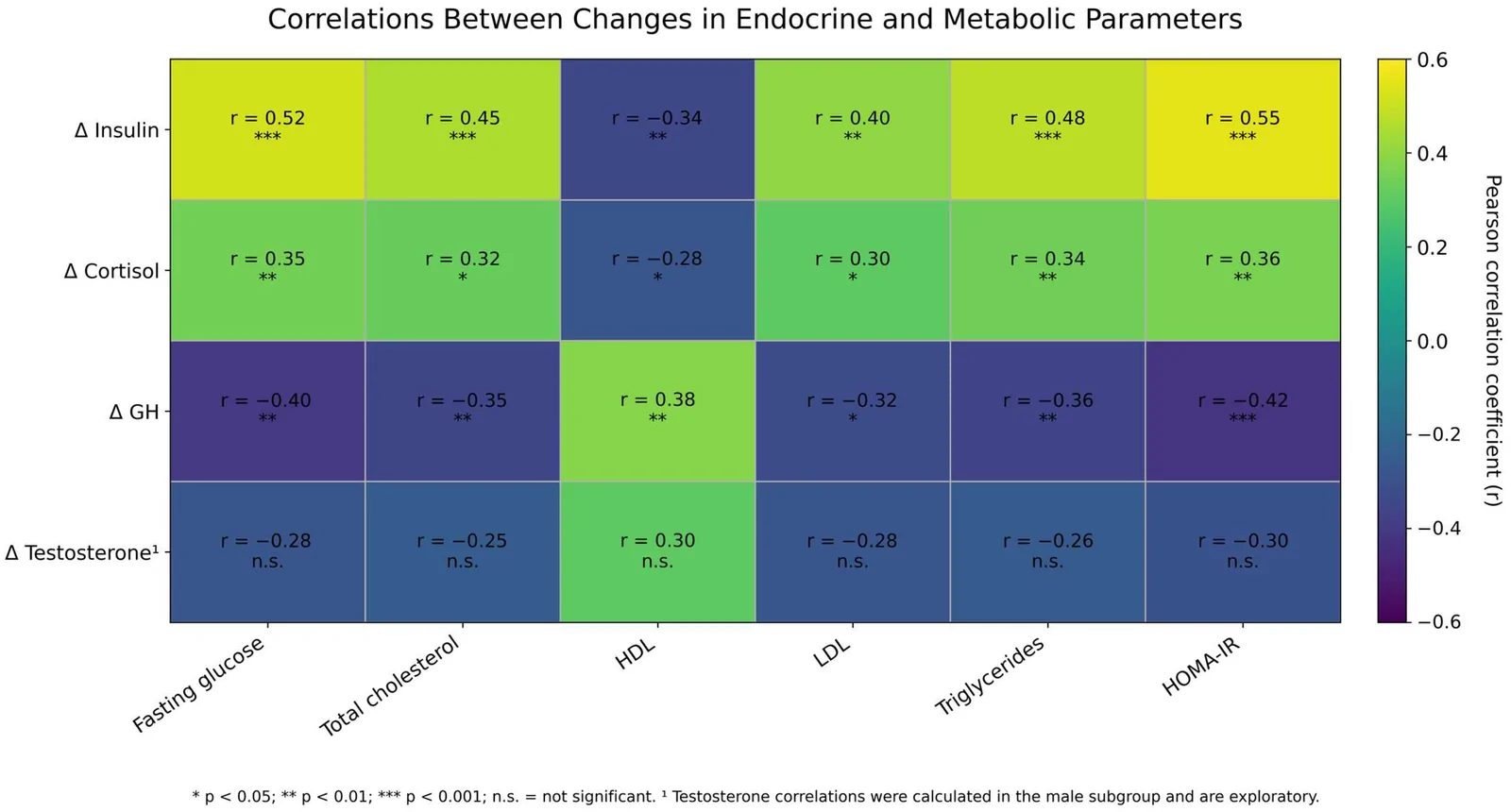
Heatmap of Pearson correlations between changes in selected endocrine and metabolic parameters.

The associations between changes in selected endocrine parameters and changes in metabolic outcomes were examined using Pearson correlation coefficients. Individual changes were calculated as the difference between post-intervention and baseline values (Δ = T1 − T0) (Table 4; Figure 2).

Changes in insulin showed the strongest associations with metabolic outcomes. Changes in insulin were positively correlated with changes in fasting glucose (r = 0.52, *p* < 0.001), total cholesterol (r = 0.45, *p* < 0.001), LDL cholesterol (r = 0.40, *p* = 0.002), triglycerides (r = 0.48, *p* < 0.001), and HOMA-IR (r = 0.55, *p* < 0.001), while an inverse association was observed with changes in HDL cholesterol (r = −0.34, *p* = 0.008). These findings indicate that greater reductions in insulin were associated with more favorable changes in glucose regulation, insulin resistance, and lipid profile, although the directionality of these relationships cannot be established from the present analyses. Changes in cortisol were positively associated with changes in fasting glucose (r = 0.35, *p* = 0.006), total cholesterol (r = 0.32, *p* = 0.013), LDL cholesterol (r = 0.30, *p* = 0.020), triglycerides (r = 0.34, *p* = 0.008), and HOMA-IR (r = 0.36, *p* = 0.005), whereas an inverse association was observed with changes in HDL cholesterol (r = −0.28, *p* = 0.030).

Changes in GH were inversely associated with changes in fasting glucose (r = −0.40, *p* = 0.002), total cholesterol (r = −0.35, *p* = 0.006), LDL cholesterol (r = −0.32, *p* = 0.013), triglycerides (r = −0.36, *p* = 0.005), and HOMA-IR (r = −0.42, *p* < 0.001), while a positive association was observed with changes in HDL cholesterol (r = 0.38, *p* = 0.003). Correlations involving total testosterone were examined separately in the male subgroup and are reported as exploratory analyses in Table 4.

## 4. Discussion

The present study evaluated the effects of a combined 12-week aerobic and resistance exercise program on the endocrine and metabolic structure of normal weight or mildly overweight adults. In particular, changes in indicators involved in endocrine regulation, including insulin, glucagon, cortisol, GH, thyroid hormones and sex hormones, as well as changes in glucose homeostasis, insulin sensitivity and lipid profile, were examined. Overall, the main results showed, in the group subjected to the combined exercise program, a favorable trend in insulin regulation, characterized by a reduction in fasting insulin and the HOMA-IR index, a decrease in baseline cortisol levels, an increase in GH and, in the male subgroup, in total testosterone. At the same time, improvements in fasting blood glucose and lipid profile were observed, with a reduction in total cholesterol, LDL and triglycerides and an increase in HDL. The observed associations between changes in insulin, cortisol, and GH and changes in metabolic outcomes also indicate a concurrent pattern of endocrine and metabolic adaptation [21]. Importantly, large Time × Group effects were observed for several key outcomes, including fasting insulin (ηp^2^ = 0.204), GH (ηp^2^ = 0.243), fasting glucose (ηp^2^ = 0.159), and HOMA-IR (ηp^2^ = 0.143). These findings indicate that the observed group differences were characterized not only by statistical significance but also by substantial effect magnitudes within the context of the present study. However, the magnitude of these physiological changes should not be interpreted as evidence of clinical benefit, as the present study was not designed to assess clinical outcomes or clinically meaningful thresholds.

One of the most relevant results of the study concerns the improvement of glucose regulation and insulin sensitivity. The reduction in fasting insulin, accompanied by the decrease in blood glucose and the HOMA-IR index, indicates favorable changes in glucose homeostasis and insulin sensitivity following the combined exercise intervention [22,23]. However, because the present study did not include aerobic-only or resistance-only comparison groups, these findings do not allow for conclusions regarding whether combined exercise provides greater metabolic adaptations than either exercise modality performed alone. From a physiological point of view, this adaptation can be interpreted in the light of the increase in peripheral glucose uptake by skeletal muscle and the improvement in metabolic efficiency resulting from regular exercise [24]. In fact, muscle contraction promotes the translocation of glucose transporter 4 (GLUT-4) to the plasma membrane through the activation of AMP-activated protein kinase (AMPK) and calcium-dependent signaling pathways, increasing muscle glucose uptake also through mechanisms partially independent of insulin [25]. These processes are associated with chronic adaptations, such as increased oxidative capacity and mitochondrial function, which can further contribute to the improvement of systemic insulin sensitivity. The results of the present study are therefore consistent with the evidence that identifies regular exercise as a central strategy for improving glycemic control and preventing metabolic alterations associated with insulin resistance [26].

A second significant result is the reduction in baseline cortisol levels observed after 12 weeks of exercise. This variation may reflect an adaptation of the regulation of HPA axis in response to repeated exposure to the exercise stimulus. Although acute exercise, especially when characterized by high intensity or duration, can result in a transient increase in cortisol, regular and progressive exposure to physical stimulus can modify neuroendocrine reactivity and promote more efficient regulation of stress response. In fact, the literature has shown that chronic training can be associated with changes in the HPA axis response and better regulation of physiological stress. In this perspective, the reduction in baseline cortisol observed in the EX-group could represent an indicator of neuroendocrine adaptation to regular exercise [27]. However, since the present study evaluated cortisol under basal conditions and not the dynamic response of the HPA axis to a standardized stressor, this result should be interpreted as a modification of the basal concentration of the hormone and not as a direct demonstration of greater resilience to stress [28].

The increase in GH levels observed in the EX-group also suggests a favorable adaptation of the somatotropic axis to the combined exercise program. GH plays an important role in the regulation of energy metabolism, lipid mobilization and tissue turnover and remodeling processes. The combination of aerobic and resistance exercise may have provided sufficient metabolic and mechanical stimulation to promote changes in the regulation of the somatotropic axis [29]. This interpretation is consistent with the literature documenting the sensitivity of the GH response to the characteristics of the training stimulus, including intensity, volume, work density and muscle mass involvement [30]. However, considering the pulsatile nature of GH secretion, the values obtained by individual samples should be interpreted with caution and considered as indicators of hormonal status under the standardized sampling conditions adopted in the study [31].

With regard to sex hormones, the increase in total testosterone observed in the male subgroup may indicate an adaptive response to the counter-resistance component of the combined program. Testosterone participates in the regulation of protein synthesis, muscle turnover and the maintenance of lean mass, and its response to exercise is influenced by the characteristics of the training program and the individual physiological state [32]. The results observed in the male subgroup are therefore consistent with the evidence regarding the modulation of anabolic hormones in response to resistance training [33]. However, this result must be interpreted considering the small number of the male subgroup. In the female subgroup, the changes in estradiol were smaller and did not show a clear differential pattern between the groups. Interpretation of this result requires particular caution, as estradiol levels can be affected by numerous biological factors, including age, menstrual cycle phase, and reproductive status [34,35]. Biological sex-related differences in endocrine response to exercise represent a complex area and the literature underlines the need for analyses specifically designed to consider these sources of variability [36]. Exploratory sex-based analyses provided no evidence of differential intervention-related changes between men and women in glucose and lipid outcomes; however, these findings should be interpreted cautiously given the limited sample size within the sex-specific subgroups.

Lipid profile findings indicate additional favorable metabolic adaptation associated with the intervention. The reduction in total cholesterol, LDL and triglycerides, together with the increase in HDL, suggests an overall improvement in the management and utilization of lipid substrates. Regular exercise increases the energy demand of skeletal muscle and promotes the mobilization and oxidation of fatty acids, processes that can progressively contribute to the remodeling of the lipid profile. Proposed mechanisms include modulation of hormone-sensitive lipase and increased lipoprotein lipase activity, which promote fatty acid mobilization and circulating lipoprotein metabolism, respectively [37]. These adaptations are consistent with evidence that associates regular exercise with an improvement in cardiometabolic risk and a reduction in metabolic processes involved in the development of atherosclerosis [38].

A relevant aspect of the results concerns the associations between changes in endocrine parameters and those of metabolic indicators. Changes in insulin were associated with changes in fasting glucose, total cholesterol, LDL, triglycerides, HOMA-IR, and HDL, with the direction of the correlations indicating that greater reductions in insulin were accompanied by a more favorable pattern of metabolic change. The direction of these associations suggests that improved insulin regulation may be closely related to a broader adaptation of glucose and lipid metabolism [39]. From a physiological point of view, increased insulin sensitivity of skeletal muscle can improve glucose utilization and reduce chronic exposure to high insulin concentrations, while improved mitochondrial function and oxidative capacity can promote more efficient utilization of both carbohydrates and lipids. This interpretation is consistent with studies that describe exercise as an integrated modulator of endocrine–metabolic networks and underline the interdependence between insulin sensitivity, muscle function and lipid metabolism [40].

The associations observed for cortisol and GH were also consistent with a broader pattern of concurrent endocrine and metabolic changes. Changes in cortisol showed associations in the direction of a less favorable metabolic profile, while the increase in GH was associated with favorable changes in the main metabolic indicators. These findings may reflect the interplay between stress regulation, availability of energy substrates, and exercise-induced anabolic and metabolic adaptations [41]. Although the correlational nature of these analyses does not allow them to establish causal relationships, the set of associations observed reinforces the interpretation of exercise as a systemic stimulus capable of simultaneously involving endocrine regulation, glucose homeostasis and lipid metabolism [42].

The results can also be interpreted by considering the endocrine role of skeletal muscle. Muscle contraction is not only a mechanical and metabolic stimulus; it determines the production and release of biologically active mediators, including various myokines. Among these, muscle-derived IL-6 has been associated with regulation of energy metabolism and immunometabolic effects distinct from those of IL-6 produced under conditions of chronic inflammation [43]. Although myokines were not directly measured in the present study, this physiological picture may help to interpret the simultaneous modification of several endocrine and metabolic domains. The results therefore support a view of physical exercise as an integrated biological stimulus, capable of promoting coordinated adaptations in glycemic regulation, lipid metabolism and neuroendocrine function [44,45].

The main strengths of the study include the longitudinal controlled and randomized design, the presence of a control group, the structured duration of the intervention and the simultaneous evaluation of multiple endocrine and metabolic indicators. A further element of interest is represented by the use of a combined program, including aerobic and resistance exercise, which allows us to examine a modality of intervention close to the commonly adopted recommendations for the promotion of metabolic health. The temporal standardization of blood samples and fasting conditions is also an important methodological element to limit part of the pre-analytical variability of hormonal and metabolic parameters. Finally, the analysis of associations between endocrine and metabolic changes offers an integrated perspective on possible systemic adaptations associated with the intervention.

However, some limitations must be considered. First, voluntary recruitment may have introduced a selection bias, favoring the participation of individuals who are more motivated or interested in the benefits of physical activity. Second, the duration of 12 weeks, although sufficient to observe short-term endocrine–metabolic adaptations, does not allow us to establish the persistence of the effects or to evaluate any plateau or regression phenomena after the conclusion of the intervention. The overall size of the sample also limits the possibility of carrying out in-depth stratified analyses, in particular for sex hormones, for which the analyses were necessarily conducted separately in the male and female subgroups. Although menstrual cycle phase was standardized between T0 and T1 at the individual level, residual biological variability in estradiol concentrations related to other reproductive factors cannot be completely excluded. In addition, sleep duration and quality before blood collection were not specifically assessed and may have contributed to residual biological variability. Moreover, the evaluation of hormones characterized by pulsatile secretion or by significant circadian variability through single blood samples, even if collected in standardized temporal conditions, does not allow for a complete characterization of their secretory dynamics. Finally, correlational analyses describe associations between variations in the variables considered, but do not allow them to establish causal relationships or mechanistic directions between endocrine and metabolic adaptations. Furthermore, the study population consisted of generally healthy adults who were normal weight or mildly overweight; therefore, the present findings should not be extrapolated to individuals with diagnosed endocrine or metabolic disorders, in whom physiological responses to exercise may differ.

In light of these aspects, future studies should include larger and more heterogeneous samples by age, biological sex and baseline metabolic status, in order to improve the generalizability of the results and allow for adequately sized stratified analyses. Follow-up of at least 6 to 12 months could help clarify the persistence of endocrine and metabolic adaptations and the possible long-term remodeling of the HPA axis, somatotropic axis and insulin regulation. Further studies should also directly compare different exercise modalities, such as high-intensity interval training (HIIT), continuous endurance, isolated resistance training and combination programs, as well as different training intensities and volumes. Particular attention should be paid to differences related to biological sex and the variables that influence the response of sex hormones to exercise. Finally, the integration between exercise programs and personalized nutritional strategies could represent an area of particular interest to understand how the interaction between training stimulus, energy availability and diet composition can modulate endocrine and metabolic adaptations.

## 5. Conclusions

The results of the present study indicate that a combined 12-week aerobic and resistance exercise program was associated with favorable endocrine and metabolic adaptations in generally healthy adults who were normal weight or mildly overweight. Specifically, the intervention was associated with changes in glucose regulation and insulin sensitivity, evidenced by reductions in fasting insulin, blood glucose, and HOMA-IR index, as well as decreased baseline cortisol levels and increased GH. In the male subgroup, an increase in total testosterone was also observed, while in the female subgroup the changes in estradiol were more contained. In parallel, the improvement of the lipid profile, characterized by the reduction in total cholesterol, LDL and triglycerides and the increase in HDL, suggests a favorable metabolic adaptation associated with combined exercise.

The observed associations between changes in endocrine parameters and those in metabolic indicators support the hypothesis that exercise-induced adaptations involve interconnected physiological processes related to insulin regulation, energy substrate metabolism, and neuroendocrine function. These results are consistent with the model according to which physical exercise acts as a systemic stimulus capable of simultaneously influencing different components of endocrine and metabolic homeostasis.

Overall, the results reinforce the role of structured exercise as a non-pharmacological strategy for the promotion of endocrine–metabolic health and suggest the potential utility of combined aerobic and counter-resistance programs in the prevention of metabolic alterations associated with insulin resistance and cardiometabolic risk. Although the results cannot be directly generalized to populations with overt metabolic diseases, they help to support the importance of regular physical activity in preventive strategies aimed at the adult population.

These findings should be interpreted as physiological adaptations within the population studied and should not be considered evidence of clinical benefit or directly generalized to individuals with endocrine or metabolic disorders. Future studies involving larger and more diverse samples, including clinical populations, and longer follow-up periods are needed to determine the clinical relevance and applicability of these adaptations, assess their persistence over time, investigate potential differences in response according to biological sex, age, and baseline metabolic status, and identify optimal combinations of exercise intensity, volume, and modality for promoting endocrine and metabolic adaptations.

## Figures and Tables

**Table 1:** Demographic and baseline characteristics of the participants.

Variable	EX (*n* = 30)	CON (*n* = 30)
Age (years)	35.2 ± 10.1	34.8 ± 9.9
Sex (M/F), *n*	15/15	14/16
BMI (kg/m^2^)	24.3 ± 3.1	24.5 ± 3.0
Habitual physical activity (MET-hours/week)	8.5 ± 2.0	8.3 ± 2.1

Note: Continuous data are expressed as mean ± standard deviation. Sex is reported as absolute frequency. EX = exercise group; CON = control group; BMI = body mass index.

**Table 2:** Changes in endocrine parameters in the EX and CON groups.

Parameter	EX T0	EX T1	CON T0	CON T1	Between-Group Difference in Change (95% CI)	F	*p*	ηp^2^
Insulin (μU/mL)	12.5 ± 4.2	8.9 ± 3.5	12.7 ± 4.1	12.4 ± 4.0	−3.3 (−4.8 to −1.8)	14.82	<0.001	0.204
Glucagon (pg/mL)	80.2 ± 15.4	78.5 ± 14.8	81.0 ± 15.6	80.8 ± 15.5	−1.5 (−6.1 to 3.1)	0.41	0.525	0.007
Total T3 (ng/mL)	1.1 ± 0.2	1.2 ± 0.2	1.1 ± 0.2	1.1 ± 0.2	0.10 (0.01 to 0.19)	4.76	0.033	0.076
Total T4 (μg/dL)	7.5 ± 1.2	7.8 ± 1.1	7.6 ± 1.1	7.6 ± 1.1	0.30 (−0.27 to 0.87)	1.12	0.294	0.019
Cortisol (μg/dL)	16.2 ± 4.3	14.5 ± 3.9	16.1 ± 4.2	16.0 ± 4.1	−1.6 (−2.9 to −0.3)	5.89	0.018	0.092
GH (ng/mL)	1.5 ± 0.4	2.3 ± 0.5	1.5 ± 0.4	1.5 ± 0.4	0.80 (0.43 to 1.17)	18.64	<0.001	0.243
Total testosterone, men (ng/dL)	450 ± 120	520 ± 130	460 ± 110	455 ± 115	75 (0 to 150)	4.21	0.050	0.135
Estradiol, women (pg/mL)	60.5 ± 15.3	65.2 ± 15.8	61.0 ± 15.1	60.8 ± 15.0	4.9 (−3.7 to 13.5)	1.36	0.253	0.046

Note: Data are expressed as mean ± standard deviation. F, *p*, and ηp^2^ refer to the Time × Group interaction. T0 = baseline assessment; T1 = assessment after 12 weeks; EX = exercise group; CON = control group; GH = growth hormone. For total testosterone: EX, *n* = 15; CON, *n* = 14. For estradiol: EX, *n* = 15; CON, *n* = 16. Sex hormones were analyzed separately within the respective biological sex subgroups.

**Table 3:** Changes in metabolic parameters in the EX and CON groups.

Parameter	EX T0	EX T1	CON T0	CON T1	Between-Group Difference in Change (95% CI)	F	*p*	ηp^2^
Fasting glucose (mg/dL)	98.2 ± 10.1	90.5 ± 9.3	97.8 ± 9.9	97.2 ± 10.0	−7.1 (−11.3 to −2.9)	10.96	0.002	0.159
Total cholesterol (mg/dL)	200.5 ± 25.3	185.2 ± 22.8	198.0 ± 24.9	196.8 ± 24.7	−14.1 (−24.5 to −3.7)	6.84	0.011	0.105
HDL cholesterol (mg/dL)	48.5 ± 10.2	52.1 ± 10.5	49.0 ± 10.0	48.8 ± 10.1	3.8 (0.4 to 7.2)	5.21	0.026	0.082
LDL cholesterol (mg/dL)	120.3 ± 20.4	110.2 ± 18.7	119.8 ± 19.9	119.0 ± 20.1	−9.3 (−16.9 to −1.7)	5.77	0.020	0.090
Triglycerides (mg/dL)	150.2 ± 35.6	130.5 ± 30.2	148.0 ± 34.9	147.2 ± 34.8	−18.9 (−32.5 to −5.3)	7.92	0.007	0.120
HOMA-IR	3.1 ± 1.2	2.5 ± 1.0	3.2 ± 1.3	3.1 ± 1.2	−0.50 (−0.82 to −0.18)	9.68	0.003	0.143

Note: Data are expressed as mean ± standard deviation. F, *p*, and ηp^2^ refer to the Time × Group interaction. T0 = baseline assessment; T1 = assessment after 12 weeks; EX = exercise group; CON = control group; HOMA-IR = Homeostatic Model Assessment for Insulin Resistance.

**Table 4:** Correlations between changes in selected endocrine and metabolic parameters.

Endocrine Change	Fasting Glucose	Total Cholesterol	HDL	LDL	Triglycerides	HOMA-IR
Δ Insulin	r = 0.52; *p* < 0.001	r = 0.45; *p* < 0.001	r = −0.34; *p* = 0.008	r = 0.40; *p* = 0.002	r = 0.48; *p* < 0.001	r = 0.55; *p* < 0.001
Δ Cortisol	r = 0.35; *p* = 0.006	r = 0.32; *p* = 0.013	r = −0.28; *p* = 0.030	r = 0.30; *p* = 0.020	r = 0.34; *p* = 0.008	r = 0.36; *p* = 0.005
Δ GH	r = −0.40; *p* = 0.002	r = −0.35; *p* = 0.006	r = 0.38; *p* = 0.003	r = −0.32; *p* = 0.013	r = −0.36; *p* = 0.005	r = −0.42; *p* < 0.001
Δ Testosterone ^1^	r = −0.28; n.s.	r = −0.25; n.s.	r = 0.30; n.s.	r = −0.28; n.s.	r = −0.26; n.s.	r = −0.30; n.s.

Note: r = Pearson correlation coefficient; Δ = T1 − T0; GH = growth hormone; HOMA-IR = Homeostatic Model Assessment for Insulin Resistance; n.s. = not significant. Correlations for insulin, cortisol, and GH were calculated in the overall sample (N = 60). ^1^ Correlations involving total testosterone were calculated exclusively in the male subgroup and should be considered exploratory.

## Data Availability

The datasets generated and analyzed during the current study are available from the corresponding author upon reasonable request.
